# Open versus laparoscopically-assisted oesophagectomy for cancer: a multicentre randomised controlled phase III trial - the MIRO trial

**DOI:** 10.1186/1471-2407-11-310

**Published:** 2011-07-23

**Authors:** Nicolas Briez, Guillaume Piessen, Franck Bonnetain, Cécile Brigand, Nicolas Carrere, Denis Collet, Christophe Doddoli, Renaud Flamein, Jean-Yves Mabrut, Bernard Meunier, Simon Msika, Thierry Perniceni, Frédérique Peschaud, Michel Prudhomme, Jean-Pierre Triboulet, Christophe Mariette

**Affiliations:** 1 Department of Digestive and Oncological Surgery, University Hospital, Place de Verdun, Lille, F-59037, France; 2 Faculty of Medicine Henri Warembourg, University of Lille 2, Lille, F-59045, France; 3 Biostatistic and Epidemiological Unit, EA 4184, Centre Georges François Leclerc, 1 rue du Pr Marion, Dijon, F-21079, France; 4 Department of Digestive Surgery, University Hospital, Avenue Molière, Strasbourg, F-67200, France; 5 Department of Digestive Surgery, Purpan University Hospital, Place du Dr Baylac, Toulouse, F-31059, France; 6 Department of Digestive Surgery, Pessac University Hospital, Avenue de Magellan Bordeaux, F-33604, France; 7 Department of Digestive Surgery, Nord University Hospital, Chemin des Bourrely, Marseille, F-13915, France; 8 Department of Digestive Surgery, University Hospital, Boulevard Montalembert, Clermont-Ferrand, F-63003, France; 9 Department of Digestive Surgery, Croix-Rousse University Hospital, Grande Rue de la Croix Rousse, Lyon, F-69004, France; 10 Department of Digestive Surgery, Pontchaillou University Hospital, Rue Henri Le Guilloux, Rennes, F-35033, France; 11 Department of Digestive Surgery, Louis Mourrier University Hospital, Rue des Renouillers, Colombes, F-92701, France; 12 Department of Digestive Surgery, Institut Mutualiste Montsouris, Boulevard Jourdan, Paris, F-75014, France; 13 Department of Digestive Surgery, Ambroise Paré University Hospital, Boulevard Charles de Gaulle, Boulogne Billancourt, F-92104, France; 14 Department of Digestive Surgery, University Hospital, Place du Pr Robert Debré, Nîmes, F-30029, France

**Keywords:** oesophageal cancer, surgery, minimally invasive surgery, oesophagectomy, randomised controlled trial

## Abstract

**Background:**

Open transthoracic oesophagectomy is the standard treatment for infracarinal resectable oesophageal carcinomas, although it is associated with high mortality and morbidity rates of 2 to 10% and 30 to 50%, respectively, for both the abdominal and thoracic approaches. The worldwide popularity of laparoscopic techniques is based on promising results, including lower postoperative morbidity rates, which are related to the reduced postoperative trauma. We hypothesise that the laparoscopic abdominal approach (laparoscopic gastric mobilisation) in oesophageal cancer surgery will decrease the major postoperative complication rate due to the reduced surgical trauma.

**Methods/Design:**

The MIRO trial is an open, controlled, prospective, randomised multicentre phase III trial. Patients in study arm A will receive laparoscopic-assisted oesophagectomy, i.e., a transthoracic oesophagectomy with two-field lymphadenectomy and laparoscopic gastric mobilisation. Patients in study arm B will receive the same procedure, but with the conventional open abdominal approach. The primary objective of the study is to evaluate the major postoperative 30-day morbidity. Secondary objectives are to assess the overall 30-day morbidity, 30-day mortality, 30-day pulmonary morbidity, disease-free survival, overall survival as well as quality of life and to perform medico-economic analysis. A total of 200 patients will be enrolled, and two safety analyses will be performed using 25 and 50 patients included in arm A.

**Discussion:**

Postoperative morbidity remains high after oesophageal cancer surgery, especially due to major pulmonary complications, which are responsible for 50% of the postoperative deaths. This study represents the first randomised controlled phase III trial to evaluate the benefits of the minimally invasive approach with respect to the postoperative course and oncological outcomes in oesophageal cancer surgery.

**Trial Registration:**

NCT00937456 (ClinicalTrials.gov)

## Background

Surgery is viewed as the best treatment for resectable thoracic oesophageal cancer (OC) with a 5-year survival rate of around 40% in patients resected with a curative intent [1,2]. Oesophagectomy using the abdominal and right-thoracic approach (Ivor-Lewis procedure) is considered to be the standard technique for middle- and lower-third thoracic OC, which represent the most frequent locations for OC, allowing for optimal loco-regional control and long-term survival. However, despite advances in surgical and perioperative management, postoperative morbidity, especially pulmonary complications, remains high and is reportedly between 30 and 50%, with a significant postoperative mortality between 2 and 10% [1].

Minimally invasive oesophagectomy (MIO) has been attempted during the last decade with the aim of improving the postoperative outcomes without compromising oncological outcomes. MIO corresponds to a collection of techniques that combine thoracoscopic and/or laparoscopic approaches, including hybrid MIO (HMIO) (laparoscopy/thoracotomy or laparotomy/thoracoscopy) and total MIO (laparoscopy/thoracoscopy). These methods have had controversial impacts on the postoperative course, and few data are available regarding the oncological outcomes [3-5].

To date, no randomised trials have been published, and the few retrospective comparative cohort studies that are available are limited by small sample sizes [6-8], major selection biases that make it impossible to compare study groups [9] and/or an absence of a rigorous definition of complications.

The thoracoscopic approach has received greater attention than laparoscopy. In the systematic reviews that have been published, thoracoscopic resections were found to have marginal benefits over open resections, such as reduced blood loss and transfusion rates and a shorter hospital stay. However similar morbidity profiles were observed, especially regarding pulmonary complications, which are the main postoperative complications after oesophagectomy [3,4]. A multicentre randomised trial (the TIME Trial) has recently been designed to study the impact of MIO (thoracoscopy and laparoscopy versus thoracotomy and laparotomy) in OC surgery, but multiple surgical procedures are proposed and there is an absence of perioperative care standardisation [10].

Laparoscopy has not been widely studied, although this technique may offer several advantages, including a lower rate of pulmonary complications (due to the less invasive nature of the procedure and reduced deterioration of the ventilatory mechanism than is observed after the open procedure [11]), ease of performance and reproducibility in specialised and non-specialised centres, the absence of laparoscopic tumoural dissection and the consequent applicability to a large number of patients regardless of the tumoural stage or neoadjuvant treatment and a lower risk of impairment of the oncological outcomes.

Several retrospective studies have suggested that HMIO with laparoscopic gastric mobilisation is feasible in OC, but these studies have major flaws, such as non-comparable groups, a small number of enrolled patients, the lack of a control group and neither pulmonary complications nor long-term outcomes considered as primary endpoints [12-16].

Because the vast majority of published studies have placed a higher priority on demonstrating feasibility than evaluating the impact on patients, and because of inconclusive results regarding the benefits of MIO with respect to outcomes, we aim to test the hypothesis that HMIO based on laparoscopic gastric mobilisation and open thoracotomy decreases major postoperative morbidity without compromising oncological outcomes through a large prospective randomised controlled trial.

## Methods/Design

### 1- Protocol overview (Figure 1)

**Figure 1:**
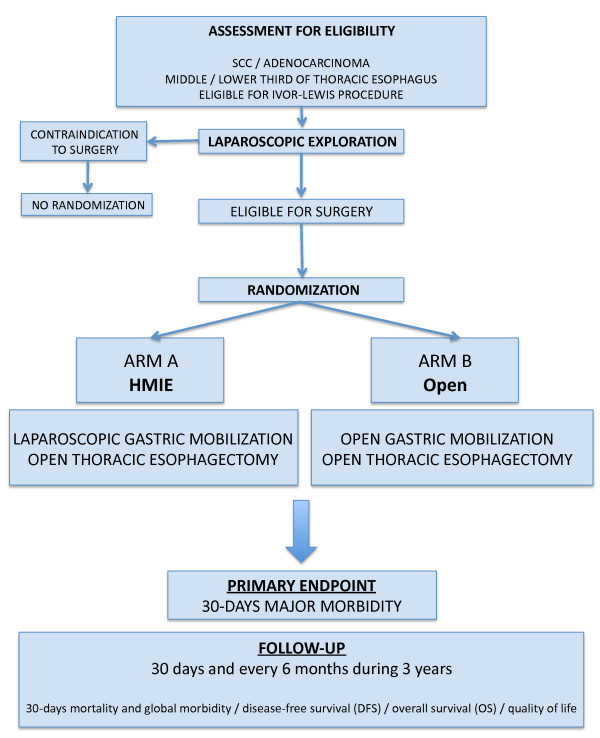
**Study flow chart**.

The MIRO trial is a prospective multicentre controlled randomised phase III trial comparing HMIO (with laparoscopic gastric mobilisation and open thoracotomy) and open oesophagectomy (with open gastric mobilisation and thoracotomy) in patients with thoracic OC who undergo oesophagectomy through the abdominal and right-thoracic approach (Ivor-Lewis procedure). In both arms of the study, patients will undergo an open-right thoracic approach. After a complete preoperative work-up and according to French national guidelines, patients who are found to be eligible for an Ivor-Lewis procedure (staged I, II or III, i.e., T1,T2,T3, N0 or N1, M0) with or without neoadjuvant treatment will be considered eligible for inclusion in this study. The surgical procedure will be scheduled 4 to 6 weeks after the completion of the neoadjuvant treatment, or within 4 weeks following the preoperative work-up in cases of primary surgery. Randomisation will be performed during the surgical procedure after a laparoscopic exploration of the abdominal cavity to confirm the absence of any contra-indication for resection. Patients in arm A will undergo HMIO, and patients in arm B will undergo traditional open oesophagectomy.

The primary endpoint is the major morbidity rate occurring within 30 postoperative days, the two groups will be compared regarding the postoperative course and oncological outcomes.

Our primary hypothesis is that HMIO will reduce major postoperative morbidity compared to the open approach.

This study is planned for a 5-year duration with a 2-year inclusion period and a 3-year follow-up period for the last included patient. The results for the primary endpoint will be available 1 month after the end of the inclusion period.

### 2- Inclusion criteria

This study will include patients with squamous cell carcinoma (SCC) or adenocarcinoma (ADC) of the middle or lower third of the oesophagus and who are eligible for surgery.

The inclusion criteria are as follows: (i) patients with SCC or ADC of the thoracic oesophagus staged I, II or III (T1, T2, T3, N0 or N1, M0) before any treatment; (ii) patients with OC in the middle or lower third or junctional Siewert's type I tumour; (iii) patients who are undergoing or not undergoing neoadjuvant radiotherapy and/or chemotherapy; (iv) patients with tumours deemed to be resectable with a curative intent at the preoperative evaluation; (v) patients who are over 18 and under 75 years of age; (vi) patients with WHO status performance of 0, 1 or 2; (vii) patients who provide a signed written consent form; (viii) patients who can undergo one of the surgical modalities to be investigated; and (ix) patients who will be available for follow-up.

### 3- Exclusion criteria

All patients who do not meet all the inclusion criteria will be excluded. The other exclusion criteria are the common contraindications for surgery related to patient status, disease extension or operative technique.

The patient-associated exclusion criteria are patients with the following features: (i) PaO2 < 60 mmHg, (ii) Pa CO2 > 45 mmHg, (iii) FEV1 < 1000 ml/sec, (iv) cirrhosis, (v) myocardial infarction or evolutive coronary artery disease, (vi) Leriche-Fontaine at stage II or more peripheral arterial occlusive disease, (vii) weight loss exceeding 15%, (viii) the presence of another malignant tumour within the last 5 years or a synchronous malignant tumour, and (ix) any other simultaneous experimental treatment.

The disease-associated exclusion criteria are (i) another histological subtype of OC besides SCC or ADC, (ii) tumours located at the pharyngoesophageal junction, the cervical oesophagus, the upper third of the oesophagus, or the oesophagogastric junction (types 2 or 3 of the Siewert's classification), (iii) distant metastases, including peritoneal carcinomatosis or metastasis to the supra-clavicular and celiac lymph nodes, (iv) recurrent nerve palsy, (v) tumoural involvement of adjacent mediastinal structures.

The surgical technique-associated exclusion criteria are (i) contra-indication for laparoscopy and (ii) a history of supra-umbilical laparotomy.

### 4- Endpoints

The primary endpoint is the major pre- and postoperative morbidity rate (occurring within 30 postoperative days). Major pre- and postoperative morbidity is defined as grade 2 (potentially life-threatening but not causing residual disability and requiring medical or invasive procedures), grade 3 (causing residual disability, including organ resection or persistence of life-threatening conditions) and grade 4 (death as a result of complications) surgical and medical complications, according to the Dindo-Clavien classification [17]. The most severe complications will be considered for the primary endpoint. However, the total number of complications per patient will also be monitored.

Secondary endpoints are postoperative mortality, postoperative overall 30-day morbidity (major and minor), the major pulmonary complication rate, disease-free survival (DFS: defined as the time interval between randomisation and the first recurrence (local, regional, distant), second cancer or death), overall survival (OS: defined as the time interval between randomisation and all deaths), quality of life (evaluated using the EORTC quality of life (QoL) questionnaires QLQ-C30 and QLQ-OES18, and EuroQol 5D) and the global economic impact of each surgical procedure.

A standardised definition of the main complications, especially pulmonary complications, are as follows:

- anastomotic leakage, defined as a symptomatic (mediastinal abscess, mediastinitis or digestive flow in chest drainage) or asymptomatic (diagnosed by water-soluble contrast swallow or digestive endoscopy) disruption of the intrathoracic anastomosis;

- gastric pull-up necrosis, defined as ischemia requiring a surgical procedure and a partial or complete removal of the gastric conduit;

- delayed gastric emptying, defined as iterative vomiting after nasogastric tube removal (from day 5), despite prokinetic treatment, requiring nasogastric tube re-placement;

- recurrent laryngeal nerve palsy;

- major postoperative pulmonary complications are defined as follows:

◦ major bronchic sputum, defined as bronchic sputum with atelectasis requiring bronchoscopy and lack of fever or hyperleukocytosis;

◦ pneumonia, defined as alveolo-interstitial radiologic infiltration with the presence of at least two of the following criteria: purulent sputum, temperature > 38.5°C or < 35°C or leukocytes > 10000/mm^3 ^or < 1500/mm^3^;

◦ respiratory insufficiency, defined as the inability of a patient to maintain a PaO2 > 60 mmHg or a PaCO2 < 55 mmHg, requiring an oro-tracheal intubation and assisted ventilation;

◦ the presence of Acute Respiratory Distress Syndrome (ARDS), defined as severe hypoxia (PaO2/FiO2 < 200), diffuse bilateral pulmonary infiltration and pulmonary wedge pressure less than 18 mmHg. Acute lung injury, defined as PaO2/FiO2 between 200 and 300, is considered ARDS.

### 5- Randomisation

Patients will be randomised during the operative procedure after performing a laparoscopic exploration of the abdominal cavity to confirm the absence of contra-indications for surgery (hepatic cirrhosis, hepatic or peritoneal metastasis, or non-resectable tumoural extension).

For patients in arm A, HMIO will be performed with a laparoscopic gastric mobilisation followed by an open thoracotomy. For patients in arm B, traditional open oesophgectomy will be performed with open gastric mobilisation through a midline laparotomy followed by an open thoracotomy.

The randomisation will be performed using the stratified block randomisation method (blocks of 4) for each centre. A randomised list will be generated for each centre and envelopes will be prepared and blinded for allocation during surgery according to serial inclusion.

### 6- Preoperative work-up

Patients with resectable OC will be considered to be operable after a complete pre-therapeutic work-up, which includes a physical examination, standard laboratory tests, an ear, nose and throat (ENT) examination, a panendoscopy under general anaesthesia and a bronchial fibroscopy with biopsies for SCC, an oesophagogastroduodenoscopy with biopsies and oesogastroduodenal barium study, an ultrasound exploration of the cervical and abdominal areas, a computed tomography (CT) scan of the thorax, mediastinum and abdomen; an oesophageal endoscopic ultrasound (EUS) examination, and a positron emission tomography if required. Clinical tumoural staging (cTNM) will be based on the data obtained from CT scan, EUS and positron emission tomography results.

### 7- Treatment methods

Perioperative procedures will be standardised independently of the approach.

#### - Global preoperative care

For all patients in the study, we will (i) treat dental, head and neck or bronchial infection, (ii) administer Immunonutrition (Oral Impact^®^, Nestlé Nutrition) 5 to 7 days prior to surgery, (iii) request cessation of smoking and drinking for at least 1 month before surgery and (iv) perform a percutaneous gastrostomy for artificial nutrition for malnourished patients (more than 10% of physical weight loss over a 6-month period).

#### - Anaesthesiology

Hydroxyzin (1 mg per kg) will be administered as a premedication 2 hours before surgery. Patients will receive standard monitoring. Two peripheral and one central venous catheters will be placed. Arterial catheter placement for continuous monitoring of the arterial blood pressure is optional. A thoracic epidural analgesia will be placed before induction of general anaesthesia. A test injection of 2 ml of xylocain with 2% adrenalin will be performed after the placement of the epidural catheter. Induction of general anaesthesia will be performed with intravenous injection of propofol (2 to 3 mg/kg), sufentanil (0.2 to 0.3 μg/kg) and cis-astracurium (0.1 to 0.15 mg/kg). Patients will be intubated with a left double-lumen endotracheal tube for single-lung ventilation during the thoracic approach. Antibioprophylaxis will be administered during induction with a second generation cephalosporin. General anaesthesia will be maintained with halogen gas (sevofluran or desfluran). Preoperative analgesia will be based on administration of 5 to 10 ml of 0.75% of ropivacain in the peridural catheter. During the thoracic wound closure, a continuous perfusion of 0.2% ropivacain (6 to 8 ml/h) will be started. Myorelaxation will be obtained with intravenous boluses of cis-atracurium (0.03 mg/kg) to maintain a maximum of two responses to the train-of-four stimulation. Protective ventilation will be performed with a small tidal volume (8 ml/kg), a respiration frequency of 10 to 15 cycles/min, an inspiration/expiration ratio of1/2, a FiO2 from 50 to 100%, and a positive end-expiratory pressure around 5 cmH20. Ventilation will be adjusted to maintain PaCO2 between 35 and 40 mmHg, venous oxygen saturation over 92% and a level of maximal pressure less than 50 cmH2O. Intravenous administration of fluids and electrolytes will be performed according to a relatively restricted regimen with 4 to 5 ml/kg/h of Ringer solution. Blood loss will be compensated by colloid administration and, if necessary, red blood cell transfusion to maintain haemoglobin levels from 7 to 10 g/dl. Early extubation will be performed at the end of the surgical procedure in normothermic patients.

#### - Surgical procedures

A laparoscopic exploration of the abdominal cavity is mandatory in the two arms, before starting the Ivor-Lewis procedure in order to confirm the absence of cirrhosis or metastatic diseases. If any contraindication to surgery is found at this time, the patient will be considered as a screen failure. Otherwise, the patient will be randomised between arm A and arm B. All patients will undergo an Ivor-Lewis procedure with the same open thoracic approach. The abdominal approach differs between the two groups: HMIO with laparoscopic gastric mobilisation will be used in arm A, and open gastric mobilisation will be used in arm B. The surgical technique has also been standardised. All patients will receive a transthoracic en bloc esophagectomy with termino-lateral anastomosis in the upper chest, including an abdominal lymphadenectomy (left and right paracardial regions along the lesser curve of the left gastric artery and celiac axis) and an extended en bloc mediastinal lymphadenectomy (left recurrent and right subclavian, paratracheal, subcarinal, left and right bronchial, lower posterior mediastinum, para-aortic, para-oesophageal lymph nodes and thoracic duct), i.e., an extended two-field lymphadenectomy. The oesophagus will be replaced by the stomach in all cases. For the laparoscopic abdominal surgical procedure, the first step is port insertion (a 10-mm optic port at an equal distance between the xyphoid and the umbilicus, a 12-mm port at the umbilicus for the stapler, a 5-mm port at the xyphoid for the retractor, two 5-mm bilateral subcostal ports and another 5-mm port in the left flank).

For the open abdominal approach, a midline supra-umbilical laparotomy will be performed.

Except for the approach, the abdominal surgical procedure will be the same in the two arms of the study. The greater curvature of the stomach will also be carefully mobilised by preserving the right gastro-epiploic arcade and dividing the short gastric vessels. The division of the gastro-hepatic ligament allows for dissection of the hiatus and the phreno-oesophageal membrane. The nodal tissue at the left gastric artery origin will be dissected en bloc with the stomach and the left gastric pedicle will be divided. The Kocher manoeuvre will be performed. Division of the gastric lesser curve by an endoscopic linear stapler will create a large gastric tube with a final staple application for this section during the thoracic step. Neither pyloromyotomy nor pyloroplasty are recommended, and a feeding tube jejunostomy will be inserted for preoperatively malnourished patients.

The thoracic approach is the same in the two arms of the study. An open thoracotomy will be performed in the 5^th ^intercostal space. An en bloc oesophagectomy will be performed after division of the inferior pulmonary ligament and division of the mediastinal pleura, up to the level of the azygos vein, which will be cut after ligation. The oesophagus, along with the perioesophageal tissue and lymph nodes, will be circumferentially mobilised with en bloc resection of the surrounding tissues and nodes as described above. The division of the oesophagus will be performed at the upper part of the chest. A frozen section analysis is mandatory before manually or mechanically performing the anastomosis. A naso-gastric tube will be placed in the gastroplasty. The chest will be closed after placement of chest drains.

#### - Postoperative care

The patients will be placed in an intensive care unit for at least 24 hours. Auto-controlled epidural analgesia will be performed with 0.2% ropivacain and 0.4 μg/ml sufentanil with continuous administration from 6 to 8 ml/h and a possibility for bolus every 20 or 30 minutes for 5 days. Analgesia will be completed using paracetamol, nefopam, tramadol, ketamine and morphinics according to the visual analogue scale. Thromboprophylaxis will begin within 6 hours following the end of the surgical procedure. During the first 24 postoperative hours, intravenous fluids will be administered (1000 to 1500 ml of 5% serum glucose). Fluid drainage quantity will be compensated with colloids volume per volume. Mean arterial pressure less than 65 mmHg or diuresis less than 0.5 ml.kg-1.h-1 will be treated by modifications of the epidural parameters, vasoactive agents or fluid administration test. In the postoperative setting, patients will receive a pulmonary physiotherapy course twice per day with incitative spirometry, non-invasive ventilation according to the PaO2 level, gastric pull-up aspiration through a nasogastric tube for 5 days, vocal cord examination, proton pump inhibitors and prokinetics. Artificial nutrition will be administered until patients are able to consume an oral diet of at least 60% of their needs with a caloric intake of 25 to 30 kcal/kg/day. Oral intake will be progressively introduced on day 6 after removal of nasogastric tube if no anastomotic leakage is suspected.

#### - Histological analysis

The following parameters will be analysed: histological subtype, tumour size, tumoural differentiation, number of analysed lymph nodes, number of involved lymph nodes, pTNM stage according the 7th edition of the TNM classification [18], vertical and lateral margins, radicality of resection (R0, R1 or R2) and tumour regression grade if neoadjuvant treatment is performed.

### 8- Data collection and follow-up

The patients will be followed-up at 30 days after surgery and every 6 months for 3 years. The follow-up protocol includes a physical examination, a thoracoabdominal CT scan every 6 months, a ENT examination once a year and a bronchoscopy every two years for SCC and a digestive endoscopy every two years. The quality of life questionnaires QLQ-30 and QLQ-OES18 will be completed by the patient at each step of follow-up during the 3 years. The EuroQol 5D is completed at 30 days and 6 months after surgery.

### 9- Participating centres

To prevent institution bias, the participating centres are required to be experienced (i) in OC surgery and (ii) in laparoscopic gastric mobilisation with at least 25 procedures performed before entering the trial. A technical surgical video has been sent to each participating centre in order to standardise surgical technique with, in selected cases, surgical technical supervision by the principal investigator (CM).

Thirteen French centres will participate in the study: the Claude Huriez University Hospital in Lille, the Croix-Rousse University Hospital in Lyon, the Ambroise Paré University Hospital in Boulogne-Billancourt, the Pessac University Hospital of Bordeaux, the Montsouris Mutualist Institute in Paris, the University Hospital of Clermont-Ferrand, the Sainte Marguerite University Hospital in Marseille, the University Hospital in Strasbourg, the University Hospital of Toulouse, the Saint-Louis University Hospital in Paris, the Louis-Mourier University Hospital Paris and the Pontchaillou University Hospital in Rennes and the University Hospital of Nîmes.

### 10- Statistical evaluation and sample size

The hypothesis of this phase III study is that the laparoscopic abdominal approach will reduce major postoperative 30-day morbidity rate. Based on published literature [3-9,11], we postulate that we will observe a reduction of major morbidity from 45% in study arm B to 25% in study arm A. To demonstrate this difference of 20% using a bilateral alpha-type-one error of 5%, a power of 80% using chi-2 and a binomial approach, the sample size required in each group is 98. Estimating that very few patients will be lost to follow-up before the 30 days follow-up required for analysis of the primary endpoint, we must include 200 patients in this study, assuming a maximum of only 4 patients lost to follow-up. The statistical analysis will be based on the intention-to-treat principle for the primary endpoint. Included patients will be analysed in their initial treatment arm as determined by treatment received and eligibility criteria. The expected inclusion duration is 2 years.

The primary endpoint will be described using frequencies and percentages with a 95% confidence interval (95% CI), and it will be compared between the groups using the Fisher exact test. Univariate and multivariate logistic regressions will be done to take into account potential cofounding effects for post-operative morbidity.

Survival endpoints will be estimated using the Kaplan Meier method and compared using the log-rank test stratified by centre. The survival endpoints will be described by their median values with a 95% CI. Follow-up will be estimated using the reverse Kaplan Meier method

Univariate and multivariate Cox analyses will be done to calculate the Hazard Ratio with 95% CI. The Fraitly model will be also used to take into account any effects of the centre.

Quality of life will be longitudinally analysed based on the time to definitive QoL deterioration [19]. The targeted dimensions will be global health, pain, alimentation, fatigue and physical functioning. Due to multiplicity, the p value for QoL will be 1%.

### 11- Ethics and safety

This study protocol was approved by the Institutional Review Board, the Nord-Ouest II ethic committee on March 2009 and the AFSSAPS (Agence Française de Sécurité Sanitaire des Produits de Santé) on May 2009 under the registration number 2009-A00144-53. The institutional promoter is the University Hospital of Lille, France. The trial has been registered on ClinicalTrial.gov website under the identification number NCT 00937456. This study received a grant from the French National Cancer Institute (INCA) in 2008.

The study complies with the Declaration of Helsinki rules and the principles of the Good Clinical Practices guidelines. Informed consent will be obtained from each patient in a written form prior to randomisation.

Patient safety and all potential threats to the patients will be monitored every 6 months by an independent data safety monitoring board (DSMB) and, additionally, at the discretion of the DSMB or Promoter. The DSMB also will evaluate the primary endpoint data. Qualified personnel at the sponsor site will also meet every three months to review safety data, including adverse events and serious adverse events. Any information deemed to potentially affect the safety of the trial will be brought to the attention of the DSMB.

## Discussion

Despite considerable improvements in OC staging, patient selection and surgical results over recent decades, the overall complication rate and the pulmonary complication rate in particular have remained high [1,19]. Therefore, it is important to develop alternative operative techniques that could achieve similar cure rates but cause less morbidity and provide a better quality of life compared with oesophagectomy with two-field lymphadenectomy based on the Ivor-Lewis procedure, which is the gold standard for treating middle- and lower-third thoracic OC.

The development of minimally invasive techniques, including laparoscopy and/or thoracoscopy, is the most important advance. The vast majority of published studies have aimed to demonstrate the feasibility of MIO more than the benefits for patients, and inconclusive results regarding the outcomes have been reported [3-5]. At the present time, there has been no randomised controlled trial evaluating the impact of MIO on postoperative and oncological outcomes. A multi-centre randomised trial (the TIME Trial) has recently been designed to study the impact of MIO compared to open oesophagectomy for cancer, but this proposal includes multiple oesophageal resection techniques, leading to potential bias [10].

We hypothesise that HMIO based on the laparoscopic procedure and an open thoracic approach may provide a significant decrease in major postoperative complications without leading to any negative impact on oncological outcomes based on: (i) the lower expected rate of pulmonary complications due to the less invasive nature of the procedure and reduced deterioration of the ventilatory mechanisms compared to the open procedure [3], (ii) the ease of performance and reproducibility at specialised and non-specialised centres, (iii) the absence of direct tumoural dissection, avoiding tumoural dissemination and (iv) applicability to a large number of patients regardless of the tumoural stage or neoadjuvant treatment administration.

For these reasons, we propose to test the hypothesis that HMIO based on laparoscopic gastric mobilisation and open thoracotomy can decrease major postoperative morbidity without compromising oncological outcomes through a large multicentre prospective randomised controlled trial. Due to the importance of perioperative and intraoperative procedures with respect to postoperative outcomes, we have standardised these techniques, as well as the definition of postoperative complications among, the participating centres.

This randomised trial may provide a high level of evidence supporting the use of HMIO in OC surgical management, as the laparoscopic procedure is considered a simple, safe, reproducible and effective technique worldwide.

## List of abbreviations

ADC: adenocarcinoma; AFSSAPS: Agence Française de Sécurité Sanitaire des Produits de Santé; ARDS: Acute Respiratory Distress Syndrome; cTNM: Clinical tumoural staging; CT scan: Computed tomography scan; DFS: Disease-free survival; DSMB: Data Safety Monitoring Board; ENT examination: Ear, throat and nose examination; EORTC: European organisation for research and treatment of cancer; EUS: Endoscopic ultrasound; FEV1: Forced expiratory volume in 1 second; HMIO: Hybrid minimally invasive oesophagectomy; INCA: French Nation Institute of Health; MIO: Minimally invasive oesophagectomy; OC: Oesophageal cancer; OS: Overall survival; QoL: Quality of Life; 95% CI: 95% Confidence interval; SCC: Squamous cell carcinoma; WHO: World Health Organization.

## Competing interests

The authors declare that they have no competing interests.

## Authors' contributions

NB wrote the manuscript; NB, GP, FB, CM were involved in the study design and assisted in writing the manuscript; FB was the statistical advisor; NB, GP, CB, NC, DC, CD, RF, JYM, BM, TP, FP, MP, JPT and CM were involved in the study design and inclusion of patients in the trial; CM is the study coordinator, obtained the grant and is responsible for the present paper; All authors read and approved the final manuscript.

## Pre-publication history

The pre-publication history for this paper can be accessed here:

http://www.biomedcentral.com/1471-2407/11/310/prepub
